# Phase II Trial of Maintenance Treatment With IL2 and Zoledronate in Multiple Myeloma After Bone Marrow Transplantation: Biological and Clinical Results

**DOI:** 10.3389/fimmu.2020.573156

**Published:** 2021-02-03

**Authors:** Rita Fazzi, Iacopo Petrini, Nicola Giuliani, Riccardo Morganti, Giovanni Carulli, Benedetta Dalla Palma, Laura Notarfranchi, Sara Galimberti, Gabriele Buda

**Affiliations:** ^1^ Hematology Unit, Department of Clinical and Experimental Medicine, University of Pisa, Pisa, Italy; ^2^ General Pathology, Department of Translational Research and New Technologies in Medicine and Surgery, University of Pisa, Pisa, Italy; ^3^ Hematology Unit and CTMO, Department of Medicine and Surgery, University of Parma, Parma, Italy; ^4^ Statistic analysis Unit, Department of Medicine and Oncology, Pisa University Hospital, Pisa, Italy

**Keywords:** myeloma, interleukin 2, transplantation, gamma delta (γδ) T cells, ZOL (zoledronic acid), maintenance therapy

## Abstract

**Background:**

Maintenance treatment after autologous bone marrow transplantation in multiple myeloma improves the outcome of patients. We designed a phase II clinical trial to evaluate the treatment with IL2 and zoledronate after autologous bone marrow transplantation in myeloma patients.

**Methods:**

Patients with a histologically proven diagnosis of multiple myeloma become eligible if achieved a very good partial remission in bone marrow samples after 3 months from autologous bone marrow transplantation. IL2 was administered from day 1 to 7. In the first cycle, the daily dose was 2 × 10^6^ IU, whereas, in subsequent ones the IL2 dose was progressively escalated, with +25% increases at each cycle, until evidence of toxicity or up to 8 × 10^6^ IU. Four mg of zoledronic acid were infused on day 2. Flow cytometry analysis of γδ-lymphocytes was performed at days 1 and 8 of treatment cycles.

**Results:**

Forty-four patients have been enrolled between 2013 and 2016. The median time to progression was 22.5 months (95% CI 9.7–35.2). A complete remission with a negative immunofixation was obtained in 18% of patients and correlated with a significantly longer time to progression (p = 0.015). Treatment was well tolerated without G3 or 4 toxicities. After a week of treatment with IL2 and zoledronate, γδ lymphocytes, Vγ9δ2, CD57+, effector, late effector, and memory γδ increased but in subsequent cycles, there was a progressive reduction of this expansion.

**Conclusions:**

The maintenance treatment with IL2 and Zoledronate has a modest activity in myeloma patients after autologous bone marrow transplantation.

**EudraCT Number:**

2013-001188-22.

## Introduction

Multiple myeloma is still a deadly disease despite relevant progress in therapy. The combination of chemotherapy with proteasome inhibitors, immunomodulatory drugs, and newly therapeutic antibodies have improved patients’ prognosis. Maintenance has been evaluated to extend duration of response in treated patients and clinical trials have begun with interferon schedule (1) followed by the more promising thalidomide (2–4) and recently lenalidomide (5–7). The activation of the immune system against residual myeloma cells is the rationale of these maintenance studies. Previously, our group has demonstrated the anti-myeloma activity of oligoclonal γδ T-cells after reduced intensity allogeneic transplantation in multiple myeloma. These results suggest a possible role of γδ lymphocytes in the eradication of the disease (8).

*In vitro*, bisphosphonates interfere with mevalonate metabolism inducing the exposition of its metabolites and modified proteins, such as CD277, on the cell surface. The treatment with bisphosphonates and IL2 can expand γδ lymphocytes and in particular the sub population harboring the Vγ9δ2 T-cell receptor (TCR) that recognize mevalonate metabolites in an MHC independent manner (9). Expanded γδ T-cells retain their ability to lysate several kinds of cancer cells lines (10). *Ex vivo* γδ lymphocytes, expanded with IL2 and bisphosphonates, efficiently kill myeloma plasma cells *in vitro* (11). IL2 and a synthetic agonist (posphostym) can expand, by one hundred-fold, γδ T-cells which retain an efficient and stable ability to kill plasma cell lines and primary human myeloma cells (12). Interestingly, γδ lymphocytes from myeloma patients do not differ from those of healthy donors in the ability to kill cancer cells *in vitro* (13). Conversely, γδ lymphocytes have an impaired cell function when obtained from patients with advanced cancers (14).

Zoledronic acid is routinely used in myeloma patients to reduce skeletal related events including fracture, pain, the necessity of radiotherapy, and hypercalcemia (15). IL2 with or without thalidomide has been evaluated for the treatment of advanced cancer in order to enhance NK activity (16). Moreover, the reinfusion of autologous IL2 activated lymphocytes has been successfully evaluated in myeloma mouse models (17). IL2 has been evaluated after autologous bone marrow transplantation to reduce minimal residual disease in lymphoproliferative disorder (18) suggesting the possibility to reach a prolonged and sustained remission in a percentage of patients.

Therefore, we decided to design a phase II clinical trial to assess the efficacy of the maintenance treatment with IL2 and zoledronate in myeloma patients after autologous bone marrow transplantation. In order to reduce the inter patient variability we decided to restrict the study to patients in very good partial remission (VGPR) after transplantation. The use of low doses of IL2 and zoledronic acid is feasible in HIV patients where increases γδ T-cells are reported (19). The maintenance treatment with lenalidomide after bone marrow transplantation is currently the standard of care in myeloma patients but when this trial has been designed data were not available yet.

## Methods

### Study Design and Participants

We designed this multicenter, single arm, phase II clinical trial in order to evaluate the maintenance treatment with IL2 and zoledronate after autologous bone marrow transplantation in patients with multiple myeloma. Patients were enrolled in the hematology units of Pisa University Hospital and Parma University Hospital after the signature of a written informed consent. The trial was conducted in accordance with the Declaration of Helsinki and in compliance with good clinical practice. The ethical boards of our institutions approved the protocol that was registered in the clinical trials information network of the Italian national observatory (EUDRACT N° 2013-001188-22).

Patients, aged between 18 and 70 years, with a histologically proven diagnosis of multiple myeloma become eligible if achieved a VGPR in bone marrow samples after 3 months from autologous transplantation, according to international myeloma working group criteria (20). Exclusion criteria include Eastern Cooperative Oncology Group performance status >2, a creatinine clearance <30 ml/min, serum calcium <8 or >12 mg/dl, osteonecrosis of the jaw, ongoing phlogosis of dental roots, and history of autoimmune or bone disorders such as Paget’s disease.

### Procedures

After the autologous bone marrow transplantation, patients that achieve a VGPR receive a maintenance treatment with subcutaneous IL-2 (Proleukin, Novartis) and intravenous zoledronic acid (Zometa, Novartis). IL2 was administered from day 1 to day 7 and in the first cycle the daily dose was 2 × 10^6^ IU. In subsequent cycles, the IL2 dose was progressively escalated, with +25% increases at each cycle, until evidence of toxicity or up to 8 × 10^6^ IU. Four mg of zoledronic acid in 100 ml of normal saline were infused on day 2. All patients received 500 mg calcium supplement and 400 IU of vitamin D daily. This schedule was repeated every 28 days until evidence of laboratory or instrumental signs of disease progression in two subsequent evaluations.

### Outcomes

Clinical evaluation was repeated every cycle. Response was evaluated according to international myeloma working group uniform response criteria (20). Response was assessed with blood tests every three cycles, with a biopsy of the bone marrow every six cycles and with ^18^fluorodesoxyglucose positron emission tomography annually or at the appearance of symptoms suggestive for disease progression. Adverse events were graded according to NCI-CTCAE v4.0.

### Blood Samples and Flow Cytometry Analysis

Peripheral Blood was collected in 5 ml EDTA tubes at cycles: 1, 2, 3, 6, 9, and 12. Blood samples were taken at day 1 of each cycle before the administration of IL2 and at day 8. The phenotype of T-cells was evaluated with the following monoclonal antibodies: CD57-FITC, PE‐γδ TCR, CD3 Per-Cp, CD45RA PE-Cy7, CD45RO, CD45APC-Cy7, CD27 V500, CD25 PE-Cy7, CD4 FITC, CD69 Per-Cp, CD3 V500, CD127 PE, CD56 APC, CD8 V500 (Becton Dickinson, San Jose, CA, USA). Corresponding irrelevant isotype‐matched mouse monoclonal antibodies were used as negative controls. Briefly, 100 μl of peripheral blood are incubated for 20 min with the respective monoclonal antibodies (7 μl for FITC and PE, 5 μl for the other fluorochromes). Then 2 ml of lysant (NH4Cl) were added for erythrocyte lysis and then centrifuged at 2.1 rpm for 5’. The resulting pellet, after removal of the supernatant, was re-suspended with 1 ml of PBS. Finally, 30 × 10^4^ total events or 10 × 10^4^ events in the lymphocyte gate were acquired on a FacsCanto II, equipped with three lasers (violet 405 nm, blue 488 nm, and red 633 nm) and analyzed with FacsDiva software (BD Bioscience, Erembodegem, Belgium).

### Statistical Analysis

The primary endpoint of the trial was the absence of disease progression after 1 year. Secondary endpoints were time to progression (TTP), progression free survival (PFS), overall survival (OS), and the toxicity profile of the treatment. Simon’s optimal two-stage design for phase II clinical trial was applied to calculate the sample size that minimizes the expected number of patients to be accrued. The sample size was calculated on a error = 0.05, b error = 0.20 assumption. P0 (clinically uninteresting true no progressive disease rate at the first re-evaluation) and P1 (sufficiently promising true no progressive disease rate after 1 year from the beginning of the treatment) were set at 75 and 90%, respectively. In the first step, at least 16 out of 20 enrolled patients without evidence of disease progression were required to continue to the second step. The trial was considered positive if 36 out 43 patients did not experience disease progression after 1 year from the beginning of the treatment.

TTP was calculated from the first day of treatment until documented disease progression; patients who did not experience disease progression were censored. PFS was calculated from the first day of treatment until documented disease progression or death; alive patients who did not experience disease progression were censored. OS was calculated from the first day of treatment until death for any causes; alive patients were censored. Kaplan-Meier curves were generated using SPSS v21.

The absolute number of γδ T-cells was calculated multiplying the number of peripheral lymphocytes/ml for the CD3+ fraction of γδ+ cells at the flow cytometer. Subpopulations of γδ and CD3 were calculated according to flow cytometer fractioning. The comparison between the number of cells at day 1 and day 8 of each cycle was performed using a paired T-test. The ratio between the number of cells at day 8 and day 1 defined the percentage of variation of cells during IL2 administration. The percentage of variation between cycles was tested using one-way ANOVA for pair data repeated measures with Dunnett’s *post hoc* tests comparing cycle 1 with each other. Statistical analysis of flow cytometry was performed using GraphPad Prism 7 (GraphPad Software, La Jolla, CA, USA). Tests were considered significant if p < 0.05.

## Results

Forty-four patients with diagnosis of multiple myeloma have been enrolled between November 2013 and November 2016. Patients’ characteristics are summarized in **Table 1**. After the initial diagnosis of multiple myeloma all patients received four cycles of bortezomib, thalidomide, and dexamethasone (VTD) followed by autologous bone marrow transplant. The transplantation was performed after a median of 9 months from the date of diagnosis. All patients enrolled in this trial obtained a VGPR and started the maintaining regimen after a median of 5 months from the day of the transplantation (range 3–9 months).

**Table 1:** Patients’ characteristics.

Patients		44	
Age		Median 60	(range 39–73)
			
Sex			
	Male	22	50%
	Female	22	50%
			
Stage			
	I	1	2%
	IIA	6	14%
	IIB	0	0
	IIIA	26	59%
	IIIB	11	25%
			
ISS			
	1	15	34%
	2	8	18%
	3	21	59%
Cytogenetic risk			
	High risk	1	5%
	Standard risk	19	95%
Immunoglobulins			
	Kappa	2	5%
	Lambda	5	11%
	IgG	33	75%
	IgA	4	9%

The median number of administered cycles was 12 (range 2–27) and 57% of patients was still on treatment at the end of the first year. The maximum tolerated dose of IL2 was 4 × 10^6^ IU in all patients but one who received 3 × 10^6^ IU. Twelve patients discontinued the treatment without evidence of disease progression because of consent withdrawal after an extensive number of cycles.

**Figure 1:**
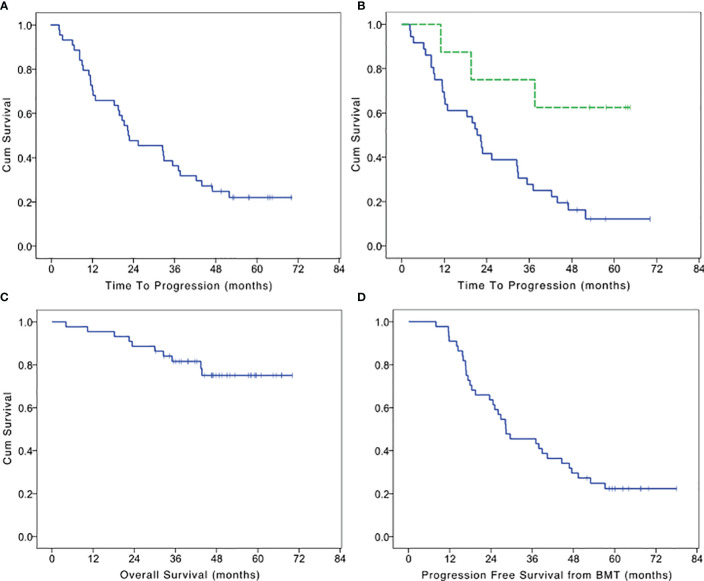
Kaplan Meier survival curves. **(A)** Time to progression of maintenance treatment with IL2 and zoledronate after autologous bone marrow transplantation in multiple myeloma patients who achieve a very good partial remission. **(B)** Time to progression in patients who achieve a negativization of immunofixation (dotted line) or not. **(C)** Overall survival of maintenance treatment with IL2 and zoledronate after autologous bone marrow transplantation in multiple myeloma patients who achieve a very good partial remission. **(D)** Progression free survival after bone marrow transplantation (BMT) in patients who achieve a very good partial remission and received maintenance treatment with IL2 and zoledronate.

At the time of data analysis, October 2019, 33 patients experienced disease progression with a median TTP of 22.5 months (95% CI 9.7–35.2 months; **Figure 1A**). PFS was identical to TTP since none of the patients died without evidence of disease progression. After 1 year from the beginning of the maintenance, 73% of patients did not experience disease progression. A complete remission with a negative immunofixation was obtained in 18% of patients (8/44) of which four maintained the response at the time of data analysis. Patients who reached negative immunofixation during maintenance treatment experienced a significantly longer TTP (LogRank p = 0.015: **Figure 1B**). With a median follow up of 48.8 months, 10 patients died. The cause of death was disease progression in all the patients. The median OS was not reached, with 95% of patients alive after 1 year from the beginning of maintenance treatment and 89% after the 2^nd^ year (**Figure 1C**). The median PFS from the autologous transplant was 28.3 months (95% CI 16.4–40.2; **Figure 1D**).

Treatment was well tolerated without G3 or 4 toxicities (**Table 2**); toxicity is summarized in **Table 1**. None of the patients discontinued the treatment because of toxicity neither requested delays of drug administration.

**Table 2:** Treatment toxicity.

	G1 (%)	G2 (%)	G3/4 (%)
**Hematological**			
Anemia	3 (7%)		
Neutropenia	3 (7%)		
**Non-hematological**			
Fever	25 (57%)	8 (18%)	
Fatigue	25 (57%)		
Arthralgia		20 (45%)	
Constipation		4 (9%)	
Nausea	5 (11%)	5 (11%)	
Cutaneous rush	2 (5%)		

### Evaluation of γδ Lymphocytes in Peripheral Blood and Bone Marrow

In *in-vitro* models, the treatment with zoledronate and IL2 expands γδ lymphocytes. Bisphosphonates interfere with mevalonate metabolisms and induce the expression of endogenous mevalonate metabolites on the surface of target cells. TCR of γδ T-cells can recognize endogenous mevalonate metabolites in an MHC independent manner (11). We demonstrated the ability of expanded γδ T-cells to kill myeloma and melanoma cell lines *in vitro* (8). Therefore, we evaluated the presence and variation of γδ lymphocytes before and during the treatment with IL-2 and zoledronate in peripheral blood and bone marrow.

**Figure 2:**
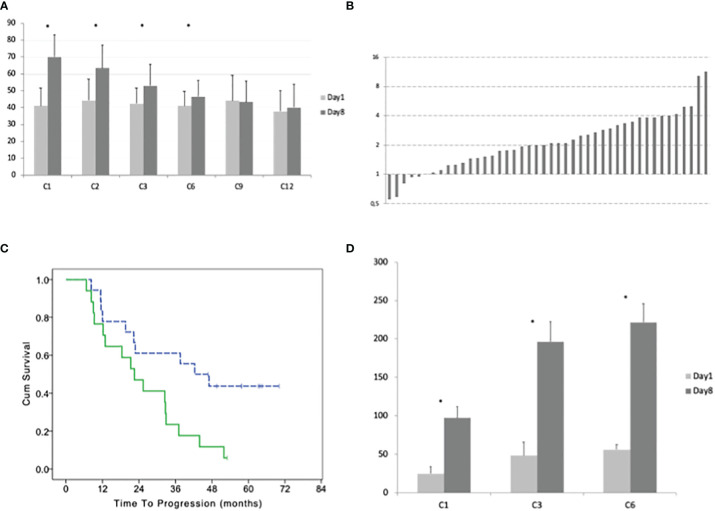
**(A)** Average with standard deviation of the number of γδ lymphocytes before the beginning and after 8 days of treatment at cycles: 1, 2, 3, 6, 9, and 12. **(B)** Ratio between the number of γδ lymphocytes at day 8 and day 1 of cycle 1 for each patient. **(C)** Time to progression of patients with a high and low (dotted line) percentage of γδ lymphocytes (according to the median value) in bone marrow before the beginning of the maintenance treatment. **(D)** Average with standard deviation of the number of CD3+ Treg lymphocytes before the beginning and after 8 days of treatment at cycles: 1, 3, and 6. Stars indicate statistically significant differences.

In peripheral blood, the median number of γδ lymphocytes was 18 cells/μl before the beginning of the treatment (range 2–356). The median number of γδ lymphocytes significantly increased after 8 days of treatment with IL2+zoledronate, with a median of 43 γδ T-cells/μl (range 2–382; paired T test p = 0.0001; **Figure 2A**).

During the first cycle of treatment, we observed an increase of γδ T-cells in 39 patients (day8/day1 ratio 2.09, range 1.02–11.39) and a decrease in 5 patients (ratio 0.80, range 0.55–0.94; **Figure 2B**). A significant difference between γδ T-cells at day 1 *vs* day 8 was observed at the 2^nd^ and 3^rd^ and 6^th^ cycles (pair t-test p = 0.004, p = 0.311, and p = 0.0237, respectively) but not at the 9^th^ and 12^th^. The ability to expand γδ T-cells by the treatment progressively reduces with subsequent cycles. Comparing the ratio between γδ T-cells at day 8 and day 1 for each cycle, we observed an overall significant difference (one-way ANOVA P < 0.0001) and with Dunnett’s *post hoc* test, we observed a significant difference between cycle 1 and cycles 3, 6, 9, and 12 but not with cycle 2.

In the bone marrow, the average percentage of γδ was 7% of CD3+ cells before the treatment (range 0.3–47%). After six cycles of therapy the average percentage of γδ/CD3 increases to 10% (range 0.8–72%) but this difference was not statistically significant.

Patients with a higher number of γδ T-cells in the bone marrow before the treatment (above the median number of 3.3%) had a worse TTP with a median of 42.3 (95% CI 23.9–60.7) *vs* 22.5 months (95% CI 13.1–31.9; LogRank p = 0.015; **Figure 2C**). There was a trend for a correlation between the number of γδ T-cells in the bone marrow and the negativization of immunofixation (Fisher exact test p = 0.052).

**Figure 3:**
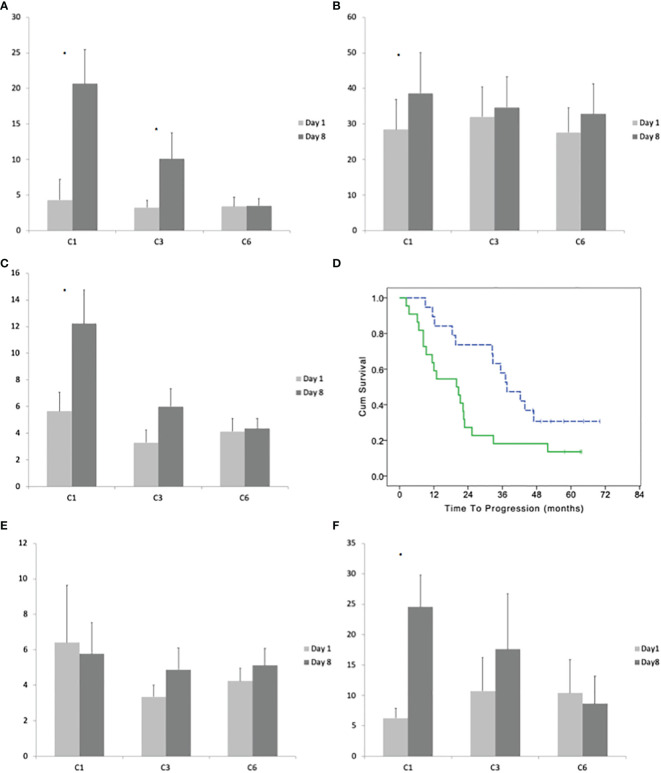
**(A)** Average with standard deviation of the number of effector (CD45RO−CD27−) γδ lymphocytes before the beginning and after 8 days of treatment at cycles: 1, 3, and 6. **(B)** Average with standard deviation of the number of late effector (CD45RO+CD27−) γδ lymphocytes before the beginning and after 8 days of treatment at cycles: 1, 3, and 6. **(C)** Average with standard deviation of the number of memory (CD45RO−CD27+) γδ lymphocytes before the beginning and after 8 days of treatment at cycles: 1, 3, and 6. **(D)** Time to progression of patients with a high and low (dotted line) number of memory γδ lymphocytes (according to their median number) in bone marrow before the beginning of the maintenance treatment. **(E)** Average with standard deviation of the number of naïve (CD45RO−CD27−) γδ lymphocytes before the beginning and after 8 days of treatment at cycles: 1, 3, and 6. **(F)** Average with standard deviation of the number of Vγ9δ2 lymphocytes before the beginning and after 8 days of treatment at cycles: 1, 3, and 6. Stars indicate statistically significant differences.

The median number of T regulatory lymphocytes (Treg) before the beginning of the treatment was 15 cells/μl (range 5–405). After 8 days of treatment, the median number of Treg significantly increased to 74 cells/μl (range 9–644; paired T-test p < 0.001). This increment did not evanish at the subsequent cycles and was even more remarkable at the 3^rd^ and 6^th^ (**Figure 2D**). Comparing the ratio between Treg at day 8 and day 1 for each cycle with one-way ANOVA, we did not observe a significant difference (F = 1.121 and p = 0.3294; **Figure 3A**).

### Variation of γδ Lymphocytes Subpopulations

CD45RO−CD27− positivity defines the T cells effector phenotype. The median number of CD45RO−CD27− γδ T-cells before the treatment was 1 cells/μl (range 0–118). After 8 days of the first cycle, the median number of CD45RO−CD27− γδ T-cells increased to 5 cells/μl (paired T-test p = 0.0015; **Figure 3A**). The ratio between day8/day1 had a median value of ratio 6.02, 2.38, and 1.09 at the 1^st^, 2^nd^, and 6^th^ cycle (One-way ANOVA p < 0.0001 with significant *post hoc* Dunnett’s test comparing cycle 1 *vs* 3 and 1 *vs* 6).

CD45RO+CD27− defines the late effector phenotype. The median number of CD45RO+CD27− γδ-cells before the treatment was 10/μl (range 1–223). After 8 days of the first cycle, the median number of CD45RO+CD27− γδ-lymphocytes increases to 14/μl (range 0–377; median ratio day8/day1 = 1.48; paired T-test p = 0.0242). There was not a significant increase in CD45RO+CD27− between day 1 and day 8 at the 3^rd^ and 6^th^ cycle (**Figure 3B**). The One-way ANOVA between cycles 1, 3, and 6 of day8/day1 ratio was not significant.

CD45RO−CD27+ defines the memory phenotype. The median number of CD45RO−CD27+ γδ T-cells before the treatment was 2/μl (range 0–36). After 8 days of the first cycle, the median number of CD45RO−CD27+ γδ T-cells increases to 5.14/μl (range 1–83; median ratio day8/day1 = 2.87; paired T-test p = 0.0090). The increase of CD45RO−CD27+ γδ T-cells at day 8 compared to day 1 was reduced in the subsequent cycles being the median ratio (day8/day1) 1.7 at the third cycle and 1.66 at 6^th^ cycle (One-way ANOVA p < 0.0120 with significant *post hoc* Dunnett’s test comparing cycle 1 *vs* cycle 3 and cycle 1 *vs* cycle 6; **Figure 3C**). The patients with lower (below the median) expansion of CD45RO−CD27+ γδ T-cells at cycle 1 had a better TTP (median TTP 37.6; 95% CI 27.8–47.4 *vs* 19.9; 95% CI: 9.2–30.6; LogRank p = 0/027; **Figure 3D**).

CD45RA+CD27+ defines the naive phenotype. The treatment with IL2 and zoledronate did not significantly increase the number CD45RA+CD27+ γδ T-cells between day 1 and day 8 of cycles 1, 3, and 6 and the one-way ANOVA between cycles was not statistically significant (**Figure 3E**).

The median number of Vγ9δ2 lymphocytes before the treatment was 2 cells/μl (range 0–58). After 8 days of treatment the median number increases to 9 cells/μl (0–152; paired T-test p = 0.0004). The increase was not significant at cycles 3 and 6 (**Figure 3F**). There was a significant difference between the ratio (day8/day1) of Vγ9δ2 cells between cycles (one-way ANOVA p = 0.0070); with Dunnett’s *post hoc* test we observed a significant difference between cycle 1 *vs* 3 and 1 *vs* 6.

**Figure 4:**
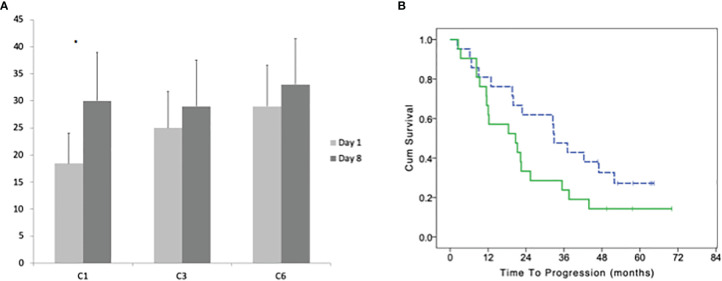
**(A)** Average with standard deviation of the number of CD57+ γδ lymphocytes before the beginning and after 8 days of treatment at cycles: 1, 3, and 6. **(B)** Time to progression of patients with a high and low (dotted line) number of CD57+ γδ lymphocytes (according to their median number) in bone marrow before the beginning of the maintenance treatment. Stars indicate statistically significant differences.

The median number of CD57+ before the treatment was 9 cells/μl (range 0–176). After 8 days of treatment the median number increases to 17 cells/μl (1–299; paired T-test p = 0.0289). The increase at day 8 *vs* day 1 was not significant at the 3^rd^ and 6^th^ cycle (**Figure 4A**). There was a significant difference between the ratio (day8/day1) of CD57+ cells between cycles (one-way ANOVA P = 0.0242); with Dunnett’s *post hoc* test we observed a significant difference between cycle 1 *vs* 3 and 1 *vs* 6. Patients with a lower increase of CD57+ cells at the first cycle (under the median value of 1.66) had a trend for a better TTP with a median of 32.9 (95% CI 26.0–39.8) *vs* 20.7 months (95% CI 7.1–34.3; LogRank p = 0.113; **Figure 4B**).

## Discussion

Our schedule of IL2 and zoledronate is feasible in myeloma patients after autologous bone marrow transplantation and results in a median TTP of 22.5 months in patients that achieved VGPR. IL2 and zoledronate expand γδ lymphocytes in peripheral blood at the first cycles but the effect reduces with subsequent administrations.

Lenalidomide has become the standard maintenance treatment after bone marrow transplantation in multiple myeloma. Indeed, a significant increase of PFS from 23 to 41 months was observed in patients treated with lenalidomide compared to placebo (6). We observed a median PFS of 28.3 months from the autologous transplantation. In subsequent update of CALGB Alliance trial, the benefit was achieved for OS in lenalidomide arm compared to placebo: 113.8 *vs* 84.1 months, respectively, despite the crossover of treatment (7). The placebo arm could represent an interesting reference for our study but lenalidomide trial includes all kinds of response, whereas our trial accrued only patients with VGPR.

Recently, a phase III trial demonstrated the superiority of ixazomib maintenance treatment after autologous stem cell transplantation in multiple myeloma with a median PFS of 26.5 *vs* 21.3 months (21). In our trial, eight patients (18%) obtained a negativization of the immunofixation during the maintenance treatment that was started at least after 3 months from the autologous transplantation suggesting an antitumor activity of the treatment. A slow clearance of the monoclonal protein can be responsible of delayed improvement of patients’ response after transplantation even without maintenance, but it usually occurs in the early months after transplant and only in a small percentage of patients. The median TTP was not reached in patients with negativization of the immunofixation, 75% of patients did not progress after 2 years from the beginning of the maintenance treatment. Adverse events, in particular fever, were manageable if IL-2 dose was limited to 4 × 10^6^ IU. Our results do not support the use of IL2/zoledronate such as standard maintenance treatment for patients with multiple myeloma that achieve VGPR after autologous bone marrow transplantation. Currently, lenalidomide remains the standard of care but patients are not definitively cured and more effective treatments are urgently needed for myeloma patients.

It is known that IL2 expand peripheral blood and tumor infiltrating T lymphocytes *in vitro* and *in vivo*. We observed an increase of γδ lymphocytes after a week of treatment with IL2 and zoledronate. This expansion includes several subpopulations of γδ lymphocytes: Vγ9δ2, CD57, effector (CD45−CD27−), late effector (CD45+CD27−) and memory (CD45−CD27+) but not naïve (CD45+CD27+) phenotypes. In the subsequent cycles, there was a progressive reduction in the ability of IL2 to expand γδ lymphocytes becoming not significant after the 6^th^ cycle. This reduction was also evident for Vγ9δ2, CD57+, effector, late effector, and memory γδ lymphocytes. A significant increment of CD3+Treg was observed at the first and subsequent cycles. Indeed, IL2 receptor is transiently expressed on the surface of lymphocytes when an antigen is recognized and help the differentiation into effector and memory T-cells. IL2 receptor is expressed also on γδ lymphocytes, and IL2 and IL15 induce the cytotoxic type 1 phenotype that produces INF-γ (22, 23). The treatment with IL2 and IL15 is not sufficient for the activation of peripheral blood γδ lymphocytes but necessitate of the activation of TCR (24, 25).

Our and other groups demonstrated that IL2 in presence of zoledronate can expand γδ lymphocytes *in vitro*. Zoledronate, interfering with mevalonate metabolisms, induces the expression of phospho-antigens such as isopentyl pyrophosphate. These antigens can be recognized in an MHC independent manner by the TCR of γδ T-cells and especially those with Vγ9δ2 rearrangements (9, 26). Phospho-antigens produce a direct activation of TCR but the understanding of how Vγ9δ2 are activated by phospho-antigens has remained a matter of speculation (27). It is known that CD277 is implicated in this process. CD277 is a surface molecule that belongs to the B7 superfamily of costimulatory proteins. Data supports the hypothesis that phospho-antigens induce CD277 secondary modifications such as joint spatial and conformational changes (27). It has been recently shown that γδ-TCR recognizes BTN2A1 and a second ligand followed by a third step involving CD277, which serves as a coactivator and interact with another receptor in a 2-receptor, 3-ligand model (28). Moreover, a large variation in antitumor reactivity of Vγ9δ2 cell clones obtained from the same or different healthy donors has been shown and only a small fraction of these clones was strongly reactive against cancer cells (39.) A highly heterogenous Vγ9δ2 T cell repertoire, with many different functional profiles and affinities of individual receptors, provides a possible explanation for the disappointing results obtained in these years using *in vitro* and *in vivo* γδ expanded lymphocytes for the treatment of hematological and solid malignancies (27). Interestingly, γδ T-cells are able to inhibit osteoclasts proliferation and restore activity of mature osteoclasts in chronic inflammatory diseases such as rheumatoid arthritis *via* interferon-γ production (26). This activity could contribute to myeloma control both by inhibiting osteolysis and inducing modification in bone marrow microenvironment. Moreover, increased doses of IL2, given at subsequent cycles, could contribute to the reduced ability to expand γδ T-cells in further administration. This may suggest that low doses of IL2 should be preferred for administration.

On the contrary, Treg constitutively express IL2 receptor and therefore remain sensitive to IL2 stimulation in subsequent cycles (29). Myeloma cells possibly contribute to the expansion of Treg *in vivo* (30). The repeated expansions of Treg could hypothetically reduce the anti-tumor activity of the treatment. However, reduced numbers of Treg carrying an impaired suppressor function have been described (31) in myeloma patients. Recently, an increase of highly reactive circulating Treg has been shown in lenalidomide treated patients (32, 33). Considering the positive results of lenalidomide maintenance in multiple myeloma the role of Treg in this setting needs to be further evaluated.

Our results suggest a role of γδ T-cells in the immune control of multiple myeloma and are consistent with our previous articles showing an increased presence of Vγ9δ2 lymphocytes in patients responding to allogeneic bone marrow transplantation (8). Several other immune cells could contribute to the anti-myeloma activity of IL2 and zoledronate treatment and our data cannot exclude their importance. Our focus on γδ T-cells depends on *in vitro* evidences of anti-myeloma activity of γδ lymphocytes that represents the rationale of this clinical trial. Patients with a lower number of γδ T-cells in the bone marrow before the maintenance treatment had a better TTP compared to those with a higher presence. Recent evidences suggest that bone marrow γδ T-cells from multiple myeloma patients are five times less cytotoxic than cells from healthy donors because of PD1 expression is induced on lymphocytes and PD-L1 in the cells of microenvironment (34). The anergy of γδ T-cells has been recently shown in B chronic lymphocytic leukemia. The reduced cytotoxicity appears to be related to the granzyme reduced secretion in effector memory cells largely expressed in chronic lymphocytic leukemia derived γδ T-cells (35). The addition of immune checkpoint inhibitors to myeloma derived γδ T-cells restore their anti-tumor activity (36).

A higher expansion of memory γδ lymphocytes (CD45RO−CD27+) was observed in patients with a worse TTP. Cytotoxic T lymphocytes with the effector phenotype (CD45RO−CD27−) are the killer of cancer cells. In acute infections, after the clearance of antigens, effector T-cells rapidly undergo apoptosis and only a subpopulation of cytotoxic memory cells survive. Memory lymphocytes are further divided in central memory and effector memory subsets, according to the expression of two homing molecules: CD62L and CCR7 (37). Effector memory cells exhibit rapid effector function, readily differentiating into highly cytotoxic effector cells (38). In contrast, central memory T-cells are less differentiated, have increased proliferative potential and acquire effector functions less rapidly. In patients with a higher number of memory γδ before treatment, there was a significant lower number of effector γδ lymphocytes (Fisher exact test p < 0.0001). A more prominent expansion of memory γδ lymphocytes was observed in patients with a higher basal number of memory γδ T-cells (chi-square p = 0.041). A prominent expansion of memory γδ lymphocytes upon IL2 stimulation could suggest the absence of antigens. Since myeloma tumor cells were still present, neither a correlation with negativization of immunofixation was observed, an inhibition of anti-tumor response could be conceivable. Also CD57+ γδ lymphocytes were expanded by IL2 zoledronate treatment *in vivo*. Patients with a higher basal number (cycle 1 day 1) of CD57+ γδ cells had a higher expansion of these cells (chi-square p = 0.001). A higher expansion of CD57+ γδ lymphocytes correlated with a worse TTP. Indeed, CD57+ identified the exhaust phenotype of effector T-cells with a reduced cytotoxicity. This further supports the idea that γδ lymphocytes can exert antitumor activity and suggests the presence of an inhibition of this phenomenon in multiple myeloma patients. Indeed, it is known that myeloma cells actively remodel the bone marrow microenvironment to establish a protective niche to evade immune control and promote cell growth (39).

In conclusion, our results suggest that IL2 and zoledronate may have activity against myeloma possibly through the activation of γδ lymphocytes. The treatment is feasible in terms of adverse events but is challenging for the patients for the numerous access to the hospital. The clinical benefit observed in terms of TTP does not support the use of maintenance treatment with IL2/zoledronate such as treatment options in myeloma patients after autologous bone marrow transplantation, especially after that lenalidomide has become the stranded of care.

## Data Availability Statement

The raw data supporting the conclusions of this article will be made available by the authors, without undue reservation.

## Ethics Statement

The studies involving human participants were reviewed and approved by the ethics committee of Pisa. The patients/participants provided their written informed consent to participate in this study.

## Author Contributions

GB was a study coordinator, wrote the paper, and managee the patients. RF and IP were study coordinators and wrote the paper. LN and BP recruited the patients. NG was a study coordinator. RM conducted the statistical analysis. GC and SG performed the laboratory tests and managed the patients. All authors contributed to the article and approved the submitted version.

## Funding

This trial has been funded with a grant from the Italian Ministry of Health: “GR-2009-1546047.”

## Conflict of Interest

The authors declare that the research was conducted in the absence of any commercial or financial relationships that could be construed as a potential conflict of interest.
